# Downregulation of Long Non-coding RNA Nuclear Paraspeckle Assembly Transcript 1 Inhibits MEG-01 Differentiation and Platelet-Like Particles Activity

**DOI:** 10.3389/fgene.2020.571467

**Published:** 2020-10-16

**Authors:** Weihua Bian, Wangping Chen, Xiaoli Jiang, Huiqing Qu, Jing Jiang, Jinfu Yang, Xinyue Liang, Bingrui Zhao, Yeying Sun, Chunxiang Zhang

**Affiliations:** ^1^School of Pharmacy, Binzhou Medical University, Yantai, China; ^2^Department of Cardiovascular Surgery, The Second Xiangya Hospital of Central South University, Changsha, China; ^3^Department of Blood Transfusion, Affiliated Hospital of Binzhou Medical University, Binzhou, China

**Keywords:** MEG-01, platelet-like particle, nuclear paraspeckle assembly transcript 1, cell differentiation, platelet activity

## Abstract

Platelets are derived from megakaryocytes and play an important role in blood coagulation. By using high throughput sequencing, we have found that the long non-coding RNA (lncRNA) nuclear paraspeckle assembly transcript 1 (NEAT1) is abundant in platelets (GEO ID: 200097348). However, little is known about its role in regulating megakaryocyte differentiation and platelet activity. This study aims to clarify the effect of NEAT1 on MEG-01 differentiation and platelet-like particle (PLP) activity. NEAT1 in MEG-01 cells was knocked down by siRNA transfection. The adhesion of MEG-01 and PLP to collagen-coated coverslips was observed under a fluorescence microscope. Flow cytometry was used to investigate cell apoptosis, cell cycle, the levels of D41/CD42b on MEG-01 cells and CD62P on PLPs. Quantitative real-time polymerase chain reaction was used to detect NEAT1 and IL-8 expression levels. Western blot was used to measure the protein levels of Bcl-2, Bax, cleaved caspase-3, and IL-8. RNA-binding protein immunoprecipitation was used to detect the interaction of NEAT1 and splicing factor proline/glutamine-rich (SFPQ). Results showed that NEAT1 knockdown decreased the adhesion ability of thrombin-stimulated MEG-01 and PLP. The expression of CD62P on PLPs and CD41/CD42b on MEG-01 cells was inhibited by NEAT1 knockdown. In addition, NEAT1 knockdown inhibited cell apoptosis with increased Bcl2/Bax ratio and decreased cleaved caspase-3, and reduced the percentage of cells in the G0/G1 phase. Meanwhile, NEAT1 knockdown inhibited the expression of IL-8. A strong interaction of NEAT1 and SFPQ, a transcriptional repressor of IL-8, was identified. NEAT1 knockdown reduced the interaction between SFPQ and NEAT1.The results suggest that lncRNA NEAT1 knockdown decreases MEG-01 differentiation, PLP activity, and IL-8 level. The results also indicate that the regulation of NEAT1 on IL-8 may be realized *via* a direct interaction between NEAT1 and SFPQ.

## Introduction

Platelets are nucleus-free fragments produced by maturemegakaryocytes (Escobar et al., 2020), which play vital roles in hemostasis, thrombosis, and inflammation (Sim et al., 2016; van der Poll and Parker, 2020). As they do not have nuclei, platelets cannot be cultured *in vitro*. Thus, the molecular mechanisms involved in platelet function are difficult to investigate. The role of long non-coding RNAs (lncRNAs) in platelets is poorly studied. However, several studies about miRNA or protein regulation on platelets utilized MEG-01 cells as the cell model (Montenont et al., 2016; Ilkan et al., 2017; Szilagyi et al., 2020). MEG-01 is a megakaryoblastic cell line that can spontaneously release platelet-like particles (PLPs) into the medium (Ogura et al., 1985). MEG-01 and PLP formation have been used as *in vitro* models for megakaryocyte and platelet related research (Kirschbaum et al., 2015; Montenont et al., 2016; Yang et al., 2016).

Long non-coding RNA nuclear paraspeckle assembly transcript 1 (NEAT1) locates in human chromosome 11 and is transcribed by RNA polymerase II. NEAT1 has two subtypes that overlap at the 5′ end, namely, NEAT1-1 with a length of 3.7 kb and NEAT1-2 with a length of 23 kb, respectively. NEAT1 plays an important role in immune response, tumor development, and prognosis *via* participating in the transcriptional regulation of other genes (Kopp and Mendell, 2018; Ghafouri-Fard and Taheri, 2019; Prinz et al., 2019; Duan et al., 2020). At present, studies on NEAT1 mainly focus on its regulatory effect on tumors. And the effects of NEAT1 on platelet-related functions have not been reported so far.

Platelets are produced by megakaryocytes through a series of differentiation processes of hematopoietic stem cells, which include megakaryocyte progenitor cell proliferation, megakaryocyte differentiation, maturation, apoptosis, and platelet release (Chen et al., 2013). NEAT1 has been shown to play an important role in promoting cell differentiation. All-trans retinoic acid could induce NEAT1 expression and promote the differentiation of the acute promyelocytic leukemia cell line NB4. The knockdown of NEAT1 by siRNA inhibited the prodifferentiation of all-trans retinoic acid in NB4 cells. This finding indicated that NEAT1 could promote the differentiation of NB4 cells (Zeng et al., 2014). In addition, NEAT1 increased during the differentiation of many kinds of cells, including embryonic stem cells, muscle cells, nerve cells, and glial cells (Lehnert et al., 2007; Mercer et al., 2010; Billing et al., 2019; Cui et al., 2019; Wang et al., 2019). NEAT1 has been shown to be highly expressed in hematopoietic stem cells, progenitors, and immune cells. Terminally differentiated cell and post-mitotic cells such as granulocytes express less NEAT1, which suggests that NEAT1 may play an important role in hematopoietic differentiation (Fallik et al., 2017). However, the regulation of NEAT1 on megakaryocyte differentiation has not been reported so far.

Nuclear paraspeckle assembly transcript 1 is an essential component in the formation and maintenance of paraspeckle (Hirose et al., 2019; Pisani and Baron, 2019), which is a protein-rich structure constructed around the lncRNA scaffold. Paraspeckels play an important role in various biological activities, such as stress response and cell differentiation (Ernst et al., 2018; Hupalowska et al., 2018; An and Shelkovnikova, 2019). Paraspeckles contain 35 proteins, which include splicing factor proline/glutamine-rich (SFPQ). SFPQ can combine with NEAT1 and also bind to an IL-8 promoter region to inhibit IL-8 transcription. NEAT1 expression is increased in cells invaded by viruses. NEAT1 shifts SFPQ from the promoter of IL-8 to paraspeckles, thereby weakening the inhibitory effect of SFPQ on IL-8 and promoting the expression of IL-8 (Imamura et al., 2014). IL-8, a member of the chemokine family, is a major inflammatory factor (Emadi et al., 2005). It can bind to its receptor on platelets, causing hyper-activation of platelets and promoting thrombosis (Bester and Pretorius, 2016). In addition, decreased IL-8 expression is associated with dysmegakaryopoiesis (Kim et al., 2019). Therefore, it was speculated that NEAT1 knockdown could decrease IL-8 expression, which might further decrease MEG-01 cell differentiation and PLP activity.

In this study, siRNA transfection was used to knock down NEAT1 and investigate the effect of NEAT1 on MEG-01 differentiation and PLP activity with the involved mechanism. The findings may provide new ideas for the treatment of platelet-related diseases.

## Materials and Methods

### Fluorescent *in situ* Hybridization

Human platelets from volunteers were extracted by a platelet extraction kit (Genmed, Shanghai, China). Fibrinogen-coated coverslips were incubated with 5% bovine serum albumin in 24-well plates for 15 min. The platelets were dropped onto the coverslips and spread at 37°C for 60 min. The platelets were then fixed with 4% paraformaldehyde for 10 min, followed by treatment with 0.5% Triton X-100 for 5 min at 4°C. After washing with phosphate buffer saline (PBS), the platelets were mixed with 0.5 μM of the Cy3 labeled-18S control and Cy3 labeled-NEAT1 fluorescent *in situ* hybridization (FISH) probe (Ribobio, Guangzhou, China), respectively, in a hybridization buffer and incubated at 37°C overnight. After washing, the coverslips were then sealed and photographed under a confocal microscope.

### Cell Culture and siRNA Transfection

MEG-01 was obtained from ATCC and cultured in RPMI 1640 medium (Gibgo, #11875119) with 10% fetal bovine serum (FBS, Gibco, #16141079). The cells were cultured in a 5% CO_2_ humidified atmosphere. For siRNA transfection assay, the cells were transfected with siNEAT1 or negative siCTRL by using RNAIMAX (#13778075, Invitrogen, CA, United States). SiNEAT1 and siCTRL were synthesized by GenePharma Co., Ltd. (Shanghai, China). The siNEAT1 sequence consisted of the sense strand GUGAGAAGUUGCUUAGAAACUUUdCdC and the antisense strand GGAAAGUUUCUAAGCAACUUCUCACUU. The sequence of the siCTRL included the sense strand UUCUCCGAACGUGUCACGUTT and the antisense strand ACGUGACACGUUCGGAGAATT.

### Reverse Transcription Quantitative Polymerase Chain Reaction

The MEG-01 cells were centrifuged and suspended in RNAiso Plus (#T9108, Takara, Otsu, Japan). The total RNA was then extracted. PrimeScript™ RT Kit (#RR047, Takara, Otsu, Japan) was used to convert RNA to cDNA. The cDNA was then amplified by quantitative polymerase chain reaction (qPCR) using SYBR Premix Ex Taq II (#RR039, Takara, Otsu, Japan). The primers for NEAT1 included the forward primer AGGCAGGGAGAGGTAGAAGG and the reverse primer TGGCATGGACAAGTTGAAGA. The primers for IL-8 included the forward primer CTTGGCAGCCTTCCTGATTT and the reverse primer ACAACCCTCTGCACCCAGTT. The 2^−ΔΔCT^ method was adopted to analyze relative ratio changes.

### Isolation of PLPs

MEG-01 cells were placed on a six-well plate with 5 × 10^5^ cells/well before transfection with siRNA. They were centrifuged 72 h after transfection at 200 × *g* for 15 min. The supernatant was transferred into a 15 ml centrifuge tube and centrifuged at 800 × *g* for 15 min. The supernatant was again transferred into another centrifuge tube and centrifuged at 1600 × *g* for 15 min. Finally, the supernatant was removed and the precipitate was the PLPs.

### Adhesion Ability of MEG-01 and PLPs

Collagen-coated coverslips were incubated with 1% bovine serum albumin in 24-well plates for 30 min. The isolated MEG-01 cells and PLPs were dissolved in cell culture medium and then stained with 1 μM DiOC6 for 15 min, respectively. The stained MEG-01 cells and PLPs were then transferred to the 24-well plate (1.5 × 10^5^ cells/well) containing collagen-coated coverslips. After incubation with 2 U/ml thrombin for 24 h, the cell culture medium was then removed. The MEG-01 cells and PLPs were washed with PBS and the coverslips were incubated with 4% paraformaldehyde for 10 min at room temperature. The coverslips were then sealed and photographed under a fluorescence microscope.

### Analysis of Platelet Activity Marker Protein CD62P

After transfection with siCTRL or siNEAT1, the PLPs were isolated and resuspended in 100 μl of PBS with 2 U/ml thrombin. The PLPs were then incubated with 20 μl of mouse anti-human CD62P (#550888, BD Biosciences, United States) for 15 min. CD62P-positive PLPs were finally detected by flow cytometry.

### Cell Counting Kit-8 Assay

MEG-01 cells were placed on a 96-well plate with 1 × 10^4^ cells/well before transfection with siRNA. After transfection for 48 h, cell viability was determined by the CCK-8 method. The cells in each well were added with 10 μl of CCK-8 solution (#CA1210, Solarbio biotechnology, Beijing, China) and then further incubated for 2 h. Absorbance at 450 nm was measured by a microplate reader. Results were presented as the percentage of values in the siCTRL group that was not treated with thrombin.

### Cell Apoptosis Analysis

MEG-01 cells were placed on a six-well plate (5 × 10^5^ cells/well) before transfection with siRNA. After transfection for 48 h, an Annexin V-FITC Kit (#556547, BD Biosciences, United States) was used to detect the apoptosis rate. In brief, the cells were centrifuged, resuspended in 100 μl of binding buffer, added with 5 μl of propidium iodide (PI) and 5 μl of Annexin V-FITC solutions, and incubated in the dark for 15 min. Finally, 400 μl of binding buffer was added and the apoptotic rate was detected by flow cytometry.

### Cell Cycle Assay

MEG-01 cells were placed on a six-well plate with 5 × 10^5^ cells/well and transfected with siRNA. After transfection for 48 h, cell cycle distribution was detected by a cell cycle kit (#KGA512, KeyGen Biotech, Nanjing, China). Briefly, the cells were first immobilized with 70% ethanol overnight at 4°C. After washing with PBS, the cells were incubated with PI/RNase A for 45 min. Finally, cell cycle distribution was analyzed by flow cytometry.

### Analysis of CD41 and CD42b

After transfection with siCTRL or siNEAT1for 48 h, MEG-01 cells were harvested and resuspended in 100 μl of PBS. The cells were then incubated with 20 μl of FITC-anti-CD41 (#MA1-19596, Invitrogen, CA, United States) and PE-anti-CD42b (#551061, BD Biosciences, United States) for 15 min. Both CD41 and CD42b-positive MEG-01 cells were finally detected by flow cytometry.

### Western Blot

The MEG-01 cells were collected and lysed with RNA-binding protein immunoprecipitation assay (RIPA) buffer (Solarbio Biotechnology, Beijing, China). After protein quantification by the BCA Protein Assay Kit (Solarbio Biotechnology, Beijing, China), equivalent amount of protein was separated by SDS-polyacrylamide gel and transferred to a PVDF membrane. The membranes were subsequently incubated with 5% milk for 1 h. Then, they were incubated with the primary antibodies overnight at 4°C, followed by the secondary antibodies at room temperature for 1 h. Finally, protein bands on the PVDF membrane were detected using an enhanced chemiluminescence detection reagent (Beyotime, Shanghai, China) and analyzed using the ImageJ software. Anti-IL-8 antibody was obtained from Absin Bioscience Inc. (#abs136518, Shanghai, China). All the other primary antibodies were purchased from Cell Signaling Technology (Beverly, MA, United States): anti-Bcl-2 (#15071), anti-Bax (#2774), anti-cleaved caspase-3 (#9654), and GAPDH (#5174).

### RNA-Binding Protein Immunoprecipitation Analysis

RNA-binding protein immunoprecipitation assay was carried out with a magna RIP Kit (#17-700, Millipore, Bedford, MA, United States). In brief, MEG-01 cells were lysed in lysis buffer for 1 h and protein A/G magnetic beads were incubated with anti-SFPQ antibody (#P2860, Sigma-Aldrich, St. Louis, MO, United States) or control anti-IgG antibody (#3420, Cell Signaling, Beverly, MA, United States) for 30 min. Subsequently, MEG-01 cell lysates were added into RIP buffer containing the magnetic bead-antibody complex and incubated for 6 h at 4°C. Purified RNA was then obtained by digesting the immunoprecipitation complex with protease K. Finally, reverse transcription quantitative polymerase chain reaction (RT-qPCR) was used to detect NEAT1 levels in the immunoprecipitation complex.

### Statistical Analysis

The GraphPad Prism 5.0 software was used to construct graphs. Results were analyzed by SPSS 19.0. All data were expressed as mean ± standard deviation (SD), and *T*-test was performed to determine differences between groups. The critical alpha level for these analyses was set at *p* < 0.05.

## Results

### NEAT1 Knockdown Decreases the Adhesion of MEG-01 Cells and PLPs

**Figure 1:**
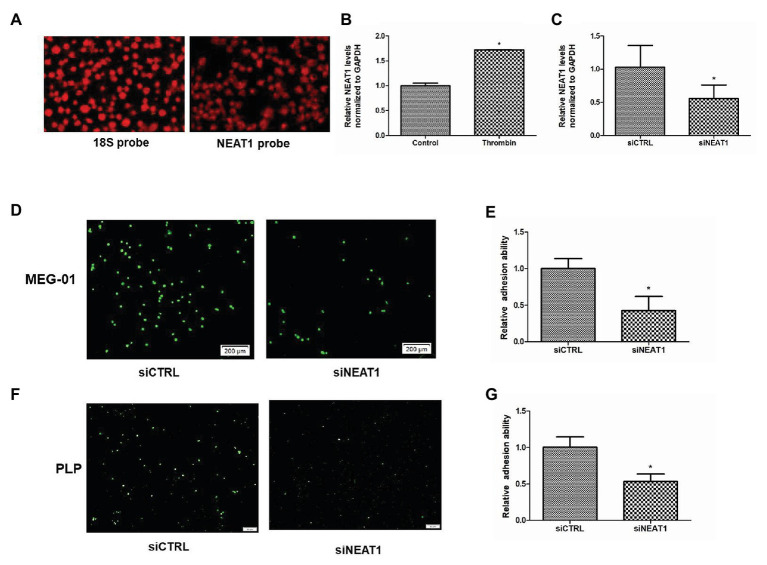
Nuclear paraspeckle assembly transcript 1 (NEAT1) knockdown decreases the adhesion ability of MEG-01 cells and platelet-like particles (PLPs). **(A)** The expression of 18S control and NEAT1 in platelets was detected by18S probe and NEAT1 probe. **(B)** NEAT1 expression was increased by thrombin treatment. MEG-01 cells were treated with 2 U/ml of thrombin for 24 h. NEAT1 levels were measured through reverse transcription quantitative polymerase chain reaction (qRT-PCR; *n* = 3, ^*^p < 0.05 vs. control). **(C)** MEG-01 cells were transfected with siCTRL or siNEAT1. NEAT1 levels were measured through qRT-PCR (*n* = 3, ^*^p < 0.05 vs. control). **(D)** MEG-01 cells were transfected with siCTRL or siNEAT1. After transfection for 72 h. The MEG-01 cells and the PLPs secreted in the medium were collected and stained with 1 mM DiOC6. The stained MEG-01 cells and PLPs were then transferred to the 24-well plate (1.5 × 10^5^ cells/well) containing collagen-coated coverslips and incubated with 2 U/ml thrombin for 24 h, respectively. MEG-01 cells adhering to collagen-coated coverslips were photographed under a fluorescence microscope. **(E)** The number of MEG-01 cells adhering to collagen-coated coverslips were analyzed using the ImageJ software. Values were expressed as mean ± SD (*n* = 3). ^*^p < 0.05 vs. siCTRL group. **(F)** Representative image of adhesion of PLPs on collagen-coated coverslips. **(G)** The number of PLPs adhering to collagen-coated coverslips were analyzed using the ImageJ software. Values were expressed as mean ± SD (*n* = 3). ^*^p < 0.05 vs. siCTRL group.

By using high throughput sequencing, we have found that the lncRNA NEAT1 is abundant in platelets (GEO ID: 200097348). In this study, FISH probe was used to detect the expression of NEAT1 in human platelets. As shown in Figure 1A, strong fluorescence intensity was observed in platelets hybridized with NEAT1 probe, which was close to that of platelets hybridized with control 18S probe. This result further confirmed that NEAT1 was abundant in platelets. Thrombin, a platelet activator, has been used to activate MEG-01 to mimic platelet activation. Thrombin can promote the activity of MEG-01 and promote their adhesion and aggregation (Montenont et al., 2016). In the current study, thrombin was used to activate MEG-01 cells. As shown in Figure 1B, the NEAT1 level was increased by 72% after thrombin treatment (*p* < 0.05). This result suggested that NEAT1 may be related to the activity of megakaryocyte and platelet.

Given that adhesion ability is an important indicator of platelet activity, the influence of NEAT1 on the adhesion of MEG-01 cells and their secreted PLPs on collagen-coated coverslips was then detected. MEG-01 cells were transfected with siRNA to knock down NEAT1. As shown in Figure 1C, relative to the siCTRL group, the siNEAT1 group showed decreased NEAT1 expression by 45% (*p* < 0.05), indicating that NEAT1 knockdown was successful. After knocking down NEAT1, the number of thrombin-stimulated MEG-01 cells adhering to collagen-coated coverslips decreased significantly (Figures 1D,E). The number of thrombin-stimulated PLPs that adhered on collagen-coated coverslips was also markedly reduced (Figures 1F,G). These results suggested that NEAT1 knockdown decreased the adhesion of MEG-01 cells and PLPs.

### NEAT1 Knockdown Decreases the Expression of the Platelet Activity Marker Protein CD62P

**Figure 2:**
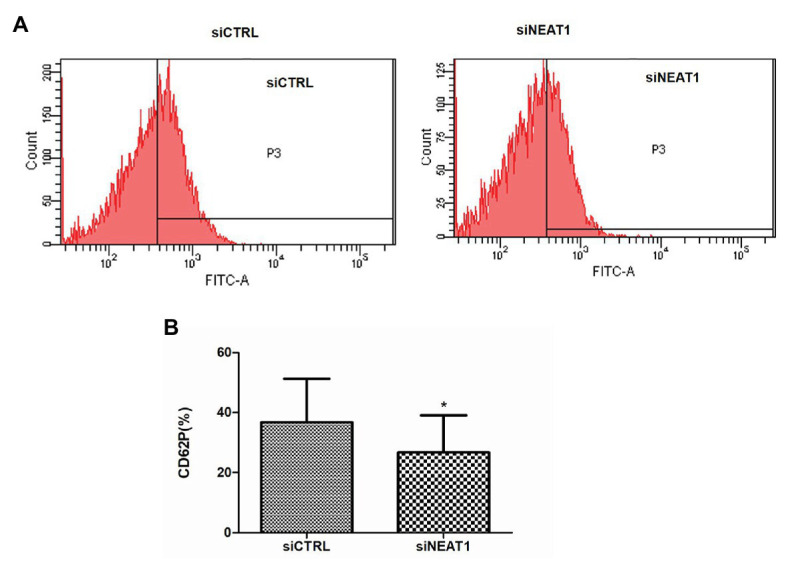
NEAT1 knockdown decreases CD62P expression on PLPs. MEG-01 cells were transfected with siCTRL or siNEAT1for 72 h. The PLPs were isolated and resuspended in 100 μl of phosphate buffer saline (PBS) with 2 U/ml thrombin, and then incubated with 20 μl of mouse anti-human CD62P (BD Biosciences, United States) for 15 min. CD62P-positive PLPs were finally detected by flow cytometry. **(A)** CD62P-positive PLPs were detected by flow cytometry. The images were representative of three independent experiments (*n* = 3). **(B)** The CD62P-positive rate was analyzed. Data were described as mean ± SD. ^*^p < 0.05 vs. siCTRL group.

CD62P is an important platelet activity marker protein on platelet surface. To investigate the effect of NEAT1 on the activity of the thrombin-stimulated PLPs, the CD62P level was detected by flow cytometry (Figure 2A). The rates of CD62P-positive PLPs in the siCTRL and siNEAT1 groups were 36.7 and 26.7%, respectively (Figure 2B). The results indicated that the PLP activity decreased after NEAT1 knockdown.

### NEAT1 Knockdown Increases the Viability and Decreases the Apoptosis of MEG-01 Cells

**Figure 3:**
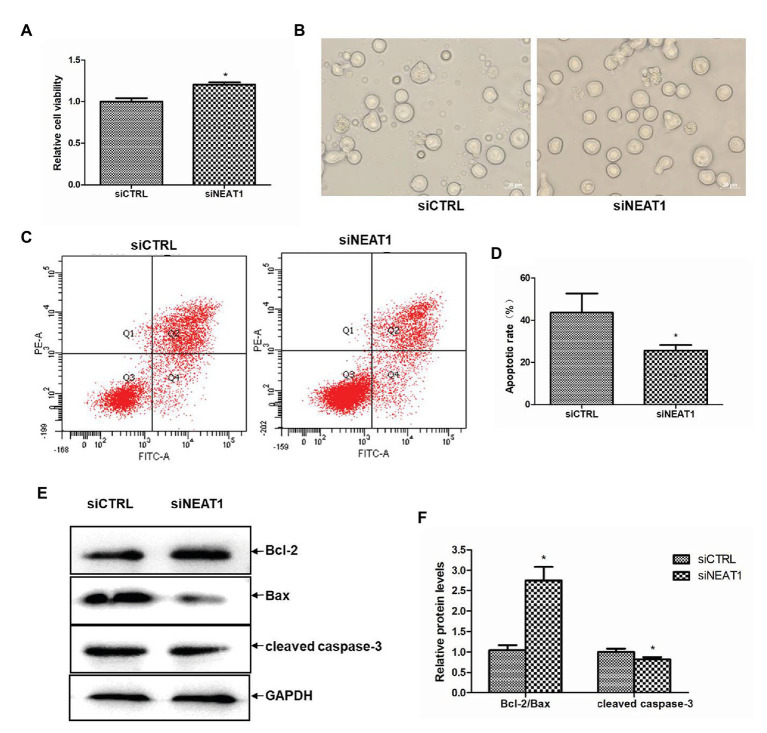
NEAT1 knockdown increases the viability and decreases the apoptosis of MEG-01 cells. MEG-01 cells were transfected with siCTRL or siNEAT1. **(A)** NEAT1 knockdown increased the viability of MEG-01 cells. The effect of siNEAT1 on the viability of MEG-01 cells was measured by CCK-8 assay (*n* = 6, ^*^p < 0.05 vs. siCTRL). **(B)** Representative microscopic images of the states of MEG-01 cells. **(C)** Annexin V-FITC/PI double staining and flow cytometry were used to detect the apoptosis rate. **(D)** The percentage sum of the lower right quadrant and the upper right quadrant was used to calculate the apoptosis rate (*n* = 3, ^*^p < 0.05 vs. siCTRL). **(E)** The expression of Bax, Bcl-2, and cleaved caspase-3 was detected by Western blot. **(F)** The Western blot results were analyzed using the ImageJ software. Values were expressed as mean ± SD (*n* = 3). ^*^p < 0.05 vs. siCTRL group.

Since PLPs are released by MEG-01-differentiated megakaryocytes, the effects of NEAT1 on the viability and apoptosis of MEG-01 cells were explored. The role of NEAT1 in the viability of MEG-01 cells was first detected by CCK-8. As shown in Figure 3A, compared with the siCTRL group, the cell viability in the siNEAT1 group was increased by 20% (*p* < 0.05). The microscopic images of the states of MEG-01 cells in siCTRL and in siNEAT1 group were shown in Figure 3B. This result suggested that NEAT1 silencing can promote the viability of MEG-01 cells.

The effect of NEAT1 silencing on cell apoptosis in MEG-01 was measured by flow cytometry (Figure 3C). As shown in Figure 3D, the apoptosis rate was 43.6% in the siCTRL group and 25.6% in the siNEAT1 group, which suggested NEAT1 silencing significantly decreased cell apoptosis. The effect of NEAT1 silencing on caspase-3 and Bcl-2 family proteins was then detected. The Western blot results are shown in Figure 3E. Relative to the siCTRL group, the siNEAT1 group showed increased level of Bcl-2/Bax by 1.75-fold and decreased level of cleaved caspase-3 by 20% (*p* < 0.05, Figure 3F).

### NEAT1 Knockdown Decreases Cell Cycle Arrest in G0/G1

**Figure 4:**
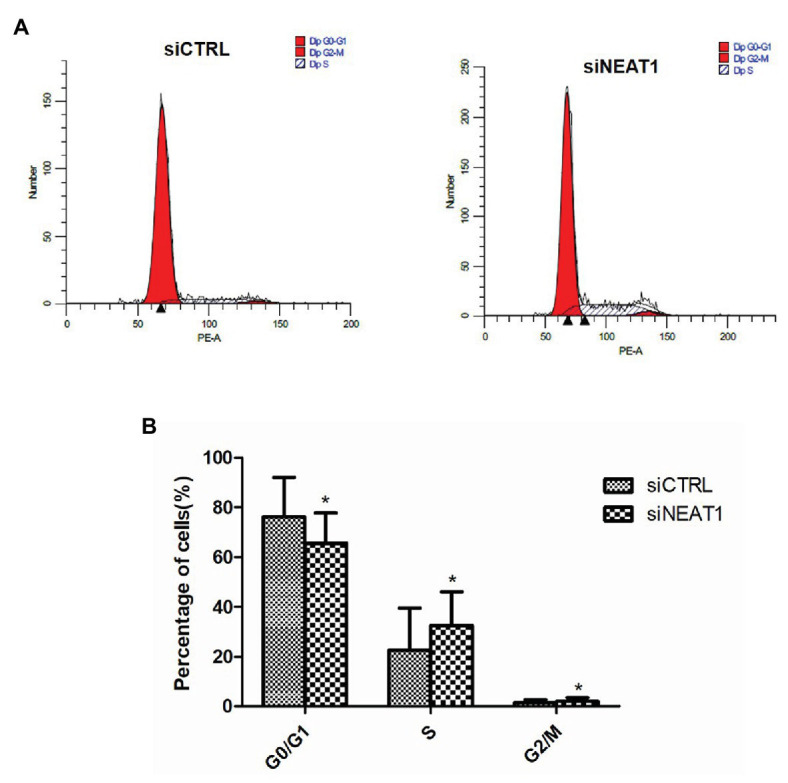
NEAT1 knockdown promotes the cell cycle progression of MEG-01 cells. MEG-01 cells were transfected with siCTRL or siNEAT1. **(A)** The cell cycle of MEG-01 cells was measured by flow cytometry. **(B)** The data for each panel came from three separate experiments and were expressed as mean ± SD (*n* = 3). ^*^p < 0.05 vs. the siCTRL group.

Considering that cell apoptosis is closely related to the cell cycle, the effect of NEAT1 silencing on the progression of cell cycle was investigated through flow cytometry (Figure 4A). The percentage of G0/G1 phase cells in the siNEAT1 group was significantly reduced relative to that in the siCTRL group (76 vs. 66%, *p* < 0.05, Figure 4B). The percentage of S phase cells was significantly increased (22 vs. 32%, *p* < 0.05, Figure 4B). These results suggested that NEAT1 knockdown could induce cell cycle progression from G1 to S phase.

### NEAT1 Knockdown Decreases Cell Differentiation

**Figure 5:**
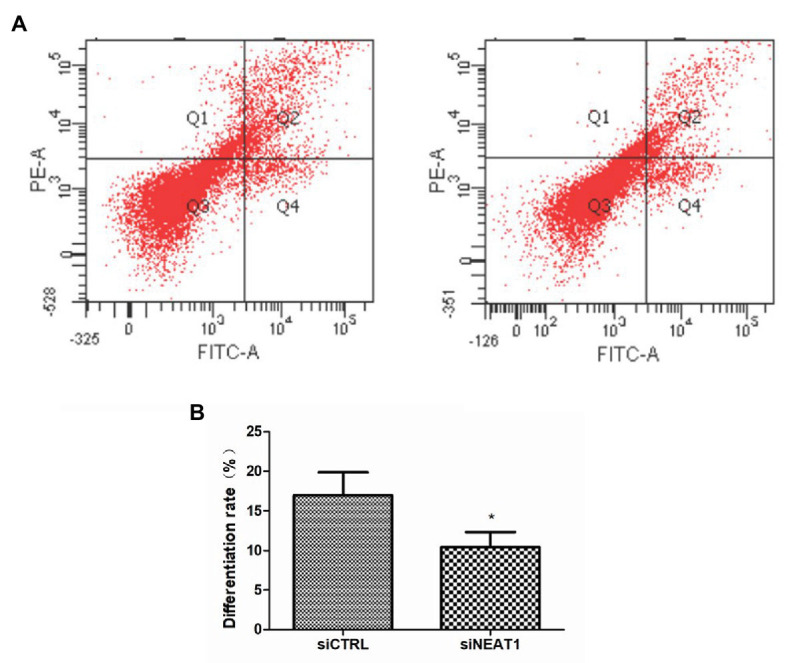
NEAT1 knockdown decreases CD41 and CD42b expression on MEG-01 cells. MEG-01 cells were transfected with siCTRL or siNEAT1 and were incubated with FITC-anti-CD41 and PE-anti-CD42b. **(A)** The CD41 and CD42b-positive PLPs were detected by flow cytometry. The images were representative of three independent experiments (*n* = 3). **(B)** The percentage of the upper right quadrant was used to calculate the differentiation rate (*n* = 3, ^*^p < 0.05 vs. siCTRL).

Considering the correlation between cell cycle and cell differentiation, the effect of NEAT1 on cell differentiation was investigated. CD41and CD42b are two markers for megakaryocyte differentiation. So, CD41 and CD42b double staining was performed on MEG-01 cells. The CD41 and CD42b levels were detected by flow cytometry (Figure 5A). As shown in Figure 5B, the positive rate for CD41/CD42b in the Q2 phase was 16.9% in siCTRL group. The positive rate was decreased to 10.4% in siNEAT1 group (*p* < 0.05, compared with the siCTRL group). These results suggested that NEAT1 knockdown could decrease MEG-01 cell differentiation, which would decrease platelet production.

### NEAT1 Knockdown Decreases IL-8 Expression in MEG-01 Cells

**Figure 6:**
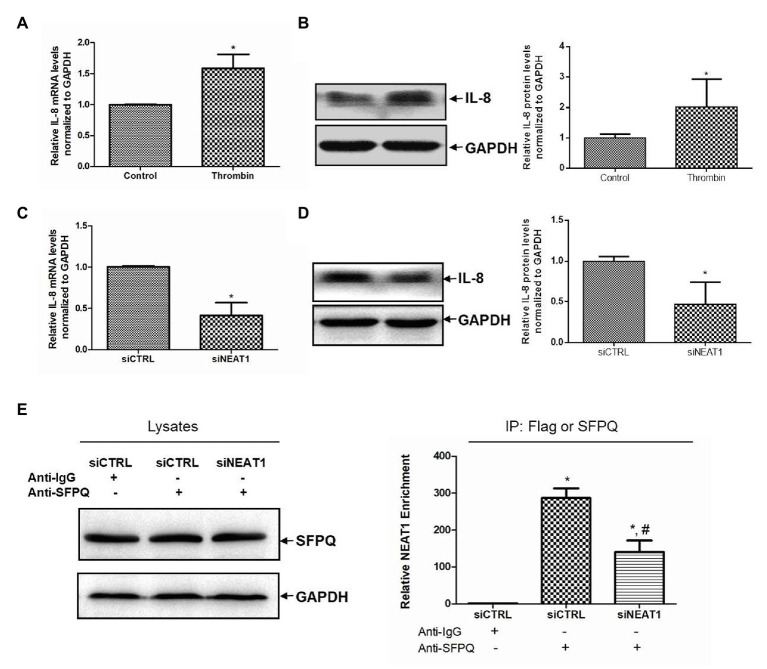
NEAT1 knockdown decreases IL-8 mRNA level in MEG-01 cells. MEG-01 cells were treated with 2 U/ml of thrombin for 24 h. IL-8 mRNA level and protein level were measured and analyzed through qRT-PCR **(A)** and Western blot **(B)**, respectively (*n* = 3, ^*^p < 0.05 vs. control). MEG-01 cells were transfected with siCTRL or siNEAT1. IL-8 mRNA level and protein level were measured and analyzed through qRT-PCR **(C)** and Western blot **(D)**, respectively (*n* = 3, ^*^p < 0.05 vs. siCTR). **(E)** MEG-01 cells were transfected with siCTRL or siNEAT1. Cells were lysed and the splicing factor proline/glutamine-rich (SFPQ) level in each group was detected by Western blot. The interaction of NEAT1 and SFPQ was verified by RIP assay. MEG-01 cell lysates containing the same amount of SFPQ were incubated with anti-IgG or anti-SFPQ antibody. NEAT1 levels in the immunoprecipitation complex were detected *via* RT-qPCR (*n* = 3, ^*^p < 0.05 vs. siCTRL + Anti-IgG; ^#^*p* < 0.05 vs. siCTRL + Anti-SFPQ).

The mechanism of NEAT1 affecting MEG-01 cell differentiation was then investigated. When thrombin induced the increase of NEAT1, it also caused the increase of IL-8 level. As shown in Figure 6A, the IL-8 mRNA level was increased by 58% after thrombin treatment (*p* < 0.05). The IL-8 protein expression level was also increased significantly in MEG-01 cells treated with thrombin (Figure 6B). In addition, NEAT1 knockdown could lead to the reduction of IL-8. As shown in Figure 6C, relative to the siCTRL group, the siNEAT1 group showed decreased IL-8 mRNA level by 59% (*p* < 0.05). As shown in Figure 6D, NEAT1 knockdown also significantly decreased the IL-8 protein level in siNEAT1 group. These results suggested that the expression trend of IL-8 was consistent with that of NEAT1. Next, the mechanism by which NEAT1 affects IL-8 expression in MEG-01 cells was studied.

Splicing factor proline/glutamine-rich is a transcriptional inhibitor of IL-8. It was attempted to further verify whether NEAT1 affected IL-8 transcription through SFPQ. The RIP experiment was carried out using anti-SFPQ and negative control anti-IgG antibody. As shown in Figure 6E, under the premise of the same SFPQ protein content in the lysate, the NEAT1 detected in the immunoprecipitation complex in anti-SFPQ group was 287-fold higher than that in the anti-IgG group. After NEAT1-knocked down, the NEAT1 detected in the immunoprecipitation complex decreased significantly. That reduction suggested that NEAT1 knockdown significantly decreased the interaction of SFPQ with NEAT1. SFPQ is known as a transcriptional inhibitor of IL-8 and can bind to the promoter region of IL-8 DNA to inhibit IL-8 transcription. These data suggest that NEAT1 knockdown may inhibit the transcription of IL-8 by reducing the binding of NEAT1 to SFPQ and increasing the binding of SFPQ to IL-8. This mechanism requires further investigation.

## Discussion

In this study, the effects of NEAT1 on MEG-01 differentiation and PLP activity were investigated *in vitro* by siNEAT1 transfection. Several valuable findings were presented in this study. Firstly, NEAT1 downregulation decreased the PLP activity. Secondly, NEAT1 downregulation reduced the differentiation of MEG-01 cells. Furthermore, NEAT1 knockdown in MEG-01 cells resulted in decreased IL-8 expression. A strong interaction was observed between NEAT1 and the transcription inhibitor SFPQ of the IL-8 gene, which may be the molecular mechanism of NEAT1 regulating IL-8 expression. It was speculated that NEAT1 might influence MEG-01 cell differentiation and PLP activity by regulating IL-8. Further study is required.

Long non-coding RNAs are a type of noncoding RNA, defined as being transcripts of more than 200 nucleotides in length, which cannot be translated into proteins (Peng et al., 2019). Excessive platelet activation is associated with an increased risk of thrombosis and there are few studies on the effects of lncRNAs on platelet activity (Zhou et al., 2019). In the current study, MEG-01 was activated with the platelet activator thrombin and it was discovered that the expression of NEAT1 was significantly increased. The NEAT1 increase suggested that NEAT1 was associated with platelet activity.

Therefore, the effect of NEAT1 on platelet activity was first explored. Under the action of thrombin and other stimulators, platelets are activated through a series of processes, such as shape change, adhesion, aggregation, and secretion. Similar to human platelets, MEG-01-derived PLPs exhibit unique microtubule organization morphology and contain several specific proteins. Those proteins play important roles in platelet function, which include CD62P and GPIIb/IIIa. Without agonists, PLPs are spherical or elliptical. After stimulation with thrombin, some PLPs become irregularly shaped with membrane protrusions and filopods. Besides the similarities in morphological changes, PLPs are also similar to human platelets in terms of thrombin-induced aggregation function and CD62P and GPIIb/IIIa expression (Takeuchi et al., 1998). Thrombin-induced MEG-01 and PLPs have been used to conduct studies on platelet activity. Montenont et al. (2016) showed that WDR1 knockdown increased thrombin-induced actin remodeling, adhesion, and spreading of MEG-01, cells as well as PLPs adhesion. The current study found that NEAT1 knockdown significantly reduced PLPs adhesion on collagen-coated coverslips and CD62P expression, which suggested that NEAT1 knockdown could reduce PLP activity.

Platelets are released by megakaryocytes which derived from a series of differentiation of hematopoietic stem cells. LncRNAs play important roles in the development and differentiation of hematopoietic stem cells. For example, lncRNA shlnc-EC6 was closely related to erythrocyte differentiation (Wang et al., 2015). LncRNA EGO played an important role in eosinopoiesis (Wagner et al., 2007). LINC00173 was associated with granulocyte differentiation (Schwarzer et al., 2017). LncRNAs are poorly investigated in megakaryocyte development field. Little attention has been paid to the role of lncRNAs in megakaryocyte differentiation and platelet activity (Dahariya et al., 2019). So far, the effect of NEAT1 on megakaryocyte differentiation has not been reported.

Caspase-directed cell apoptosis has played important roles in megakaryocyte differentiation. Both the broad-spectrum caspase inhibitors and caspase-3 inhibitors have significantly reduced the differentiation of megakaryocytes (Clarke et al., 2003; McArthur et al., 2018; Kovuru et al., 2020). Cell differentiation is also relevant to the cell cycle. Cell differentiation occurs during the G1 phase of the cell cycle and slows down when the G1 phase is short or almost non-existent (Soufi and Dalton, 2016; Asenjo et al., 2020; Ci et al., 2020). In the current study, it was found that NEAT1 knockdown increased cell viability, decreased cell apoptosis, promoted cell cycle progression, and inhibited cell differentiation. These results suggested that NEAT1 knockdown could decrease the differentiation of MEG-01 cells.

After confirming the role of NEAT1 in the differentiation of MEG-01, it was attempted to explore the potential molecular mechanism. The interleukin family plays a very significant role in inflammation. They circulate in the blood and, therefore, directly affect platelet activity. Hypercoagulation is a common feature of many inflammatory response processes (Bester et al., 2018). In the current study, in addition to NEAT1 level, IL-8 level was also increased in thrombin treated-MEG-01 cells. It was hypothesized that overexpression of NEAT1 in MEG-01 cells would induce the elevation of IL-8 expression, although NEAT1 was not artificially overexpressed in MEG-01 cells. In addition, transfection of siRNA was used to reduce the expression of NEAT1. It was found that when NEAT1 was knocked down, IL-8 expression was also reduced. The above results indicated that IL-8 was regulated by NEAT1 and the expression level of IL-8 was positively correlated with the expression level of NEAT1. These data have shown consistent results to previous studies. One example of previous studies was that the expression of NEAT1 and IL-8 was upregulated in the synoviocyte of osteoarthritis. The knockdown of NEAT1 could lead to the decrease of IL-8 (Wang et al., 2017). Other examples were seen in the blood of patients with sepsis or acute ischemic stroke, where the expression of NEAT1 was increased and positively correlated with IL-8 (Huang et al., 2018; Li et al., 2020). IL-8 has been reported to play important roles in cell differentiation. IL-8 could induce the chondrogenic differentiation of bone marrow mesenchymal stem cells *in vivo* and *in vitro* through the PI3k/AKT cell signaling pathway (Yang et al., 2018). The secretion of IL-8, chemerin, and CXCL16, by papillary renal cell carcinoma, promoted the recruitment of monocytes and the differentiation of macrophages with foam cell phenotype (Krawczyk et al., 2017). IL-8 and GM-CSF were required in the differentiation of acute monocytic leukemia induced by CD44 (Delaunay et al., 2008). Decreased IL-8 expression was associated with dysmegakaryopoiesis (Kim et al., 2019). Therefore, it was speculated that NEAT1 knockdown decreased IL-8 expression, which might further decreased MEG-01 cell differentiation and PLP activity.

RNA-binding protein immunoprecipitation assay was performed to explore the mechanism by which NEAT1 regulates IL-8 in MEG-01 cells. A previous study showed that SFPQ was a transcriptional inhibitor of IL-8 and could bind to the promoter region of IL-8 DNA to inhibit IL-8 transcription. Immune stimuli induced an increase in NEAT1 expression in HeLa TO cells. The increase of NEAT1 expression strengthened the binding of NEAT1 to SFPQ. The increased binding of NEAT1 to SFPQ decreased the binding of SFPQ to IL-8 promoter, thus promoting the expression of IL-8 (Imamura et al., 2014). Our results also showed the strong interaction between NEAT1 and SFPQ in MEG-01 cells, which was consistent with their findings. Moreover, our results showed that NEAT1 knockdown significantly decreased the interaction of SFPQ and NEAT1. It suggested that NEAT1 knockdown might lead to more binding of SFPQ to IL-8 by reducing the amount of NEAT1-bound SFPQ, thereby inhibiting IL-8 transcription. Further investigation is required.

## Conclusion

In conclusion, lncRNA NEAT1 knockdown decreased MEG-01 cell differentiation and PLP activity. NEAT1 knockdown also decreased IL-8 expression through SFPQ in MEG-01 cells. NEAT1 showed potential as a therapeutic target for platelet-related diseases. The function of IL-8 on MEG-01 cell differentiation requires further investigation.

## Data Availability Statement

The raw data supporting the conclusions of this article will be made available by the authors, without undue reservation.

## Author Contributions

CZ, YS, and WB designed the study. Most experiments were performed by WB and WC. XJ contributed to CD62P analysis assay. HQ contributed to adhesion assay. XL and BZ contributed to cell viability, apoptosis, and cell cycle experiments. JY and JJ were involved in manuscript corrections. WB and WC co-wrote the manuscript. All authors contributed to the article and approved the submitted version.

### Conflict of Interest

The authors declare that the research was conducted in the absence of any commercial or financial relationships that could be construed as a potential conflict of interest.
